# Is social identity theory enough to cover sports fans’ behavior?: additional perspective from identity fusion theory

**DOI:** 10.3389/fpsyg.2025.1574520

**Published:** 2025-06-03

**Authors:** Taeyeon Koo, Hyungi Harry Kwon, Jaeeun Shin, Juhae Baeck

**Affiliations:** ^1^Industry-Academy Cooperation Foundation, Sangmyung University, Seoul, Republic of Korea; ^2^Department of Physical Education, Chung-Ang University, Seoul, Republic of Korea; ^3^Office of Research, Chung-Ang University, Seoul, Republic of Korea

**Keywords:** social identity theory (SIT), identity fusion theory, sports fans, role identity theory, elaborated social identity model (ESIM)

## Abstract

This paper investigates the applicability of identity fusion theory (IFT) in explaining sports fan behaviors, highlighting its advantages over traditional frameworks such as social identity theory (SIT) and role identity theory (RIT). While SIT and RIT provide significant insights into group identification and role-based behavior, they fall short in addressing the profound emotional and relational dimensions of sports fandom. These limitations are particularly evident in contexts involving extreme loyalty, self-sacrificial actions, and the deep personal connections fans form with their teams and fellow supporters. IFT bridges these gaps by positing that personal and group identities can merge, creating a powerful motivational force that drives fans to prioritize the group’s welfare as their own. This paper explores how IFT’s dual focus on relational and collective ties offers a more nuanced understanding of fan loyalty, pro-group behavior, and the intense emotional investment characteristic of sports fandom. By examining the interplay of these ties, this paper provides theoretical and practical implications for advancing research on fan engagement and loyalty. The findings suggest that IFT not only complements but extends the explanatory power of SIT and RIT, offering a comprehensive framework for understanding the unique dynamics of sports fandom.

## Introduction

The study of sports fan behavior has long been an area of interest for scholars seeking to understand the psychological and social mechanisms driving fan identification, loyalty, commitment, and extreme pro-group actions. Over the years, social identity theory (SIT) and role identity theory (RIT) have emerged as dominant frameworks for explaining how individuals develop and maintain their connections to sports teams and fan communities (Kwon et al., 2022; Lock and Heere, 2017; Osborne and Coombs, 2013).

Social identity theory explains how individuals derive self-esteem and identity through membership in social groups. It accounts for behaviors like ingroup favoritism (Wann and Branscombe, 1993), Basking in Reflected Glory (BIRGing), and Cutting Off Reflected Failure (CORFing) (Dwyer et al., 2016; Cialdini et al., 1976). SIT also sheds light on intergroup dynamics, such as rivalries and outgroup derogation, which bolster ingroup identity (Branscombe and Wann, 1992). However, SIT’s focus on depersonalization—where personal identity is subsumed by group identity—has limitations. It inadequately captures the emotional and relational bonds fans share with their teams and fellow supporters. Additionally, while effective at explaining general ingroup behaviors, SIT struggles to address extreme loyalty or self-sacrificial actions, such as enduring hardships to attend games or engaging in risky behavior to support a team (Swann et al., 2012).

Role Identification Theory (RIT) complements SIT by focusing on the roles individuals assume within social contexts. In sports fandom, fans internalize roles like “supporter” or “enthusiast,” guiding their behavior based on role expectations (McCall and Simmons, 1978). Despite its strengths, RIT also has limitations. Its emphasis on individual roles often overlooks the broader social and emotional aspects of group membership. While RIT explains how fans internalize specific roles, it does not fully capture the intense unity and shared purpose that drive fans to act beyond role expectations.

Sports fandom frequently surpasses basic group identification or the fulfillment of social roles, embodying behaviors that indicate a profound personal dedication to the team and/or the community. This commitment may manifest through the willingness to incur financial and emotional expenses to support a particular team, as well as through engagement in risky activities aimed at upholding its reputation.

Traditional theories, while effective in explaining general patterns of group identification and role-based behavior, struggle to fully account for these unique and extreme forms of fan behavior (Swann et al., 2012). Identity fusion theory (IFT), a more recent conceptual framework, has emerged as a promising alternative to address these gaps. By emphasizing the emotional and relational dimensions of identity, IFT posits that individuals can experience a profound sense of oneness with a group, leading to a willingness to engage in extraordinary actions for its benefit (Swann et al., 2009).

This paper explores the application of identity fusion theory in the context of sports fandom, addressing the limitations of SIT and RIT in explaining extreme fan behaviors. By examining how relational and collective ties contribute to identity fusion among fans, this research seeks to provide a more comprehensive understanding of the psychological and social dimensions of sports fandom. The study aims to demonstrate how IFT can complement and extend traditional theories, offering new insights into fan behavior and its implications for sports organizations.

## Literature review

### Social identity theory and sports fan behavior

Social identity theory is a prominent framework in social psychology that examines how individuals define themselves based on their membership in social groups. Introduced by Tajfel and Turner (1979), SIT highlights the interplay between cognitive categorization and psychological attachment to social groups, providing a basis for understanding how individuals derive self-esteem and social identity through group affiliation. SIT has been extensively applied to various domains, including sports fandom, where it explains phenomena such as ingroup favoritism and rivalry (Branscombe and Wann, 1992), organizational behavior, where it accounts for team cohesion and workplace dynamics (Ashforth and Mael, 1989), and intergroup conflict, where it sheds light on the processes driving prejudice and discrimination (Tajfel and Turner, 1979). The theory’s emphasis on intergroup behavior and social categorization has also informed research on social cohesion in multicultural societies. These applications make SIT one of the most influential models for understanding group dynamics. This literature review outlines the foundational principles of SIT, explores its key concepts such as self-categorization and depersonalization, and examines its applications and limitations across diverse contexts of sport fandom.

Social identity theory emerged from Tajfel’s earlier research on intergroup discrimination, which demonstrated that individuals exhibit favoritism toward members of their own group (ingroup) even when the group is arbitrarily defined (Tajfel, 1970). This finding led to the development of SIT, which posits that individuals derive a significant part of their self-concept from their group memberships. Tajfel and Turner (1979) proposed that group identification is motivated by a desire to maintain or enhance self-esteem, which is influenced by comparisons between one’s ingroup and relevant outgroups.

The theory is built on three core components of social categorization, social identification, and social comparison. These components interact to shape intergroup behaviors, including ingroup favoritism, outgroup discrimination, and collective action (Tajfel and Turner, 1979). One of the central tenets of SIT is that individuals strive to maintain a positive social identity by favoring their ingroup over outgroups. This bias manifests in preferential treatment of ingroup members and derogation of outgroup members (Tajfel, 1982). In the context of sports fandom, Wann and Branscombe (1993) found that fans often express loyalty and pride in their team while simultaneously discrediting rival teams. This behavior serves to reinforce the individual’s positive identity through association with the ingroup’s success.

Social identity theory explains phenomena such as BIRGing and CORFing, where individuals publicly associate themselves with successful ingroups and distance themselves from underperforming ones. Cialdini et al. (1976) demonstrated that sports fans were more likely to wear team apparel after a win than after a loss, a behavior driven by the need to enhance self-esteem through group affiliation. This aligns with SIT’s assertion that individuals derive a sense of pride and self-worth from their group’s achievements.

Social identity theory has been widely used to explain the formation and maintenance of social cohesion within groups. Hogg and Abrams (1988) argued that strong group identification leads to greater conformity to group norms and values, enhancing group cohesion. In organizational settings, SIT has been applied to understand team dynamics, showing that employees with strong organizational identification are more likely to engage in cooperative behaviors and exhibit higher job satisfaction (Ashforth and Mael, 1989).

Social identity theory provides insights into the origins and escalation of intergroup conflict. When groups perceive threats to their distinctiveness or status, they may engage in competitive or hostile behaviors to reaffirm their identity (Tajfel and Turner, 1979). For instance, in political contexts, SIT has been used to explain the rise of nationalism and ethnocentrism, where group identity is bolstered by emphasizing differences from outgroups. Despite its widespread application, SIT is not without limitations. Critics have pointed out that the theory’s emphasis on group-based cognition often neglects individual-level variability in group identification and behavior (Hogg and Terry, 2000). For example, not all members of an ingroup exhibit the same level of loyalty or bias, suggesting that other factors, such as personal values or situational contexts, play a role.

### Role identity theory and sports fan behavior

Role identity theory (RIT) offers a framework for understanding how individuals internalize roles within social contexts and how these roles influence their behavior and self-concept. Originating from symbolic interactionism, RIT was developed by McCall and Simmons (1978) to explore how roles are negotiated, maintained, and enacted in various social environments. The theory has found wide applicability in fields such as sociology, psychology, and organizational studies, and it has been increasingly used to explain behaviors in contexts such as sports fandom, organizational participation, and social movements. This literature review discusses the foundations, key components, applications, and limitations of RIT, with a focus on its contributions to understanding identity and behaviors of sport fans.

Role identity theory is grounded in the idea that individuals derive their sense of self from the roles they occupy in social structures (McCall and Simmons, 1978). These roles are socially constructed and associated with specific expectations, norms, and behaviors. For example, roles such as parent, student, employee, or sports fan come with socially defined responsibilities and behavioral scripts. According to RIT, individuals internalize these roles, which become central components of their self-concept. The more important a role is to an individual’s self-concept, the greater the influence it exerts on their behavior.

One of RIT’s central tenets is the notion of role salience**—**the degree to which a particular role is prioritized in an individual’s hierarchy of identities. Stryker and Serpe (1982) expanded on this concept, emphasizing that individuals tend to behave in ways consistent with their most salient roles. For example, a highly salient role as a sports fan may lead an individual to prioritize attending games or engaging in team-supportive behaviors over other competing roles.

The key concepts of identity theory are role commitment and identity verification, role negotiation, and role conflict. Role commitment refers to the extent to which an individual is invested in a particular role, often measured by the time, resources, and effort dedicated to fulfilling the role’s expectations (Burke and Reitzes, 1981). Commitment to a role reinforces its salience and strengthens its influence on behavior. Additionally, the process of identity verification, wherein individuals seek feedback that confirms their role identity, plays a crucial role in maintaining the self-concept. For instance, a committed sports fan might derive validation through recognition from fellow fans or positive reinforcement for their support of the team.

Role identity theory also addresses the phenomenon of role conflict, which arises when the expectations of one role are incompatible with those of another. For instance, a sports fan who is also a parent may experience conflict when the demands of attending a child’s event clash with a team’s game schedule (Thoits, 1991). Role conflict can influence identity salience and lead to changes in role prioritization.

Role identity theory has been extensively applied to the study of sports fans, who often adopt the role of “enthusiast” or “supporter.” This role becomes central to their self-concept and influences behaviors such as attending games, wearing team merchandize, and participating in fan communities (Trail et al., 2003). Wann et al. (2001) found that fans with a highly salient sports fan role were more likely to engage in team-supportive behaviors and experience emotional highs and lows based on the team’s performance. In addition, Lock and Heere (2017) noted that RIT is, along with SIT, is one of the main theoretical grounds in sport fan behavior research.

While RIT provides valuable insights into identity and behavior, it is not without limitations. One critique is that the theory may overly focus on individual agency in role negotiation, neglecting structural and cultural constraints that limit role flexibility (Hogg et al., 1995). For example, societal norms may impose rigid expectations on certain roles (e.g., attending Ohio State University, making it difficult for an individual to be a fan of Michigan, which s/he is originally from). Additionally, RIT’s focus on individual roles may overlook the interplay between personal and collective identities, which theories like identity fusion theory address more effectively (Swann et al., 2009).

### Identity fusion theory

Identity fusion theory offers a psychological framework to understand how individuals develop a profound sense of alignment and unity with a group, which drives them to engage in extreme pro-group behaviors, including acts of self-sacrifice. This theory extends beyond traditional models such as SIT (Tajfel and Turner, 1979), by emphasizing the coexistence of personal and social identities within fused individuals. Swann et al. (2009) argue that identity fusion does not involve a depersonalization of self but rather a merging of individual and group identities, where personal and collective goals become intertwined.

A key feature of identity fusion is the presence of both relational ties (close interpersonal connections within the group) and collective ties (shared goals and values across the group), creating a deep emotional commitment to the group (Swann et al., 2012). This dual connection is critical in motivating individuals to undertake extreme actions, such as defending or sacrificing for the group (Gómez et al., 2011). Furthermore, fused individuals retain a strong sense of agency, perceiving their personal contributions as vital to the group’s welfare, which differentiates this theory from depersonalized group identification models.

Empirical studies have demonstrated the practical applications of identity fusion in explaining behaviors ranging from altruistic sacrifice to group extremism. For instance, Whitehouse et al. (2017) observed that combat veterans with fused identities were more likely to recall instances of extreme loyalty and personal risk undertaken for their comrades. Similarly, Buhrmester et al. (2018) found that Americans who identified strongly with bombing victims during the Boston Marathon attack were more likely to provide aid, reflecting the motivational power of fusion. A salient example of identity fusion in sports is the extraordinary commitment displayed by fans who undertake significant personal sacrifices to support their teams. For instance, during the 2012 FIFA Club World Cup, approximately 30,000 Corinthians fans from Brazil travelled to Japan to support their team, with many quitting their jobs or incurring substantial debts to make the journey. Such actions underscore the depth of fusion, where the team’s success is perceived as a personal triumph, motivating fans to engage in costly behaviors for the group’s benefit. Identity fusion also provides insight into the participation of some fans in collective violence. Research indicates that members of ultra groups, characterized by intense loyalty and cohesion, exhibit higher levels of identity fusion, which correlates with a greater propensity for violent behavior during matches. In studies conducted in Indonesia and Australia, incidents of violence and antisocial behavior were significantly higher among ultras compared to general fans, highlighting how fusion can drive individuals to engage in extreme actions to defend or promote their group’s interests.

#### Relational ties and collective ties in identity fusion theory: insights from sports fans

Identity fusion theory emphasizes the critical role of relational ties and collective ties in fostering a profound and enduring connection between individuals and groups. Relational ties refer to the close interpersonal bonds that individuals form within a group, while collective ties reflect the shared values, goals, and overarching identity that unify the group as a whole (Swann et al., 2012). In the context of sports fans, these dual dimensions of identity fusion manifest through deeply personal connections with other fans and a shared commitment to their team, which can drive intense loyalty and even extreme pro-group behaviors. Relational ties within identity fusion theory are characterized by intimate and emotionally charged bonds between group members. Among sports fans, these ties often emerge through regular interactions in shared spaces, such as stadiums, fan clubs, or online communities. These bonds are not merely transactional but are imbued with emotional resonance, as fans share the highs and lows of supporting their team. Research shows that such interpersonal connections can significantly enhance fans’ attachment to their teams, as their relationships with other supporters become a core part of their identity (Wann et al., 2011).

For example, studies on football (soccer) fans highlight the importance of relational ties in creating a sense of community. Dixon (2014) found that among English Premier League fans, friendships formed through regular attendance at matches were pivotal in reinforcing loyalty to their team. Fans who developed close relationships with others in the stands were more likely to exhibit behaviors such as traveling long distances to attend matches, engaging in coordinated chants, and showing unwavering support even during the team’s underperformance. These relational ties not only strengthen individual identity fusion but also foster a sense of belonging that can be critical in sustaining fan loyalty over time.

While relational ties are grounded in interpersonal connections, collective ties pertain to the shared values, goals, and collective identity of the group. Among sports fans, these ties are often rooted in the symbolic and cultural significance of their team. The shared history of the team, its iconic players, and memorable victories serve as focal points for collective identification. Fans align themselves with the team’s narrative, adopting its successes and failures as their own, which fosters a collective identity that transcends individual differences (Branscombe and Wann, 1992).

An illustrative example of collective ties among sports fans can be observed in the rituals and traditions surrounding major teams. For instance, the shared singing of anthems such as Liverpool FC’s *You’ll Never Walk Alone* creates a unifying experience for fans. This anthem, sung collectively by thousands of supporters before matches, reinforces a sense of belonging and solidarity. Research by Newson et al. (2016) highlights how such synchronized behaviors can enhance identity fusion by fostering a perception of shared purpose and commitment among fans.

#### Interplay of relational and collective ties

The interplay of relational and collective ties is critical in driving identity fusion among sports fans. Relational ties provide the emotional depth and intimacy needed for personal connection, while collective ties offer a broader framework for shared values and identity. Together, these ties create a robust psychological attachment to the group, leading to the high level of loyalty among sports fans.

For example, in studies of ultra-fan groups, relational ties are often evident in the intense friendships and camaraderie among members, while collective ties are reinforced through shared rituals, chants, and the defense of team honor (Doidge et al., 2020). These combined ties can motivate fans to engage in behaviors such as defending their team against rival fans, even at personal risk. Gómez et al. (2011) found that identity fusion rooted in both relational and collective ties was a strong predictor of extreme behaviors, including public demonstrations of loyalty and willingness to sacrifice for the group.

The role of relational and collective ties in sports fandom offers valuable insights into how identity fusion operates in real-world contexts. Understanding these dynamics is particularly relevant for sports organizations and marketers aiming to enhance fan engagement and loyalty. For instance, creating opportunities for relational ties through fan meetups, social events, and online communities can foster deeper connections among supporters. Similarly, emphasizing collective ties through storytelling, rituals, and the promotion of shared values can strengthen the overall sense of unity within the fan base.

Social identity theory, as posited by Tajfel and Turner (1979), conceptualizes group identification as a process wherein individuals categorize themselves into social groups, leading to the internalization of group norms and values. This identification fosters in-group favoritism and out-group discrimination, primarily through cognitive mechanisms such as social categorization, identification, and comparison. The emphasis is on the collective self, with individual behavior guided by group norms and the desire for positive distinctiveness.

In contrast, IFT, developed by Swann et al. (2009), introduces the concept of a visceral sense of “oneness” with the group, wherein the boundaries between personal and social identities become highly permeable. This fusion leads to a synergistic relationship between the personal and social selves, resulting in a heightened propensity for extreme pro-group behaviors, including self-sacrifice. Unlike SIT, which emphasizes the depersonalization of the self in favor of group identity, IFT underscores the simultaneous activation and mutual reinforcement of both personal and social identities.

While both theories address the relationship between individuals and groups, their focal points differ. SIT primarily accounts for behaviors driven by conformity to group norms and the pursuit of positive social identity. IFT, however, explains behaviors that transcend normative expectations, particularly in contexts requiring personal sacrifice for the group. Recognizing this, we have elaborated on scenarios where both identification and fusion may coexist, influencing behavior in complementary ways. For instance, in high-stakes situations, individuals with strong group identification may adhere to group norms, while those experiencing identity fusion may engage in extraordinary acts of self-sacrifice.

### The added value of identity fusion theory in explaining and predicting sports fan behaviors

While SIT and RIT have long been foundational frameworks for understanding sports fan behaviors, IFT offers unique advantages that extend beyond the explanatory power of these theories. By addressing the interplay between personal and group identities and emphasizing the emotional and relational aspects of group membership, IFT provides a more nuanced framework for exploring fan behaviors. This theory is particularly effective in explaining high-level loyalty and behaviors that SIT and RIT may struggle to fully capture (Newson et al., 2016).

#### Identity fusion with emotional involvement

Social identity theory focuses primarily on the cognitive and social processes associated with group membership, including ingroup favoritism and outgroup derogation (Cialdini et al., 1976; Hornsey, 2008; Tajfel and Turner, 1979). While effective at explaining group-based dynamics, SIT often emphasizes depersonalization, wherein the individual’s identity becomes subsumed under the group identity (c.f., Chun and Sagas, 2022). This approach may inadequately address behaviors rooted in a deeper emotional connection or the willingness of individuals to prioritize the group’s welfare over their own.

Role identity theory, on the other hand, centers on how specific roles shape an individual’s self-concept and behavior (McCall and Simmons, 1978). While this framework effectively explains how fans internalize the role of “supporter” or “enthusiast,” it often treats roles as relatively static constructs, overlooking the fluid and emotionally charged nature of group membership that evolves in response to situational or relational factors.

While SIT and RIT have been extensively applied in the domain of sports fan behavior, particularly in understanding team allegiance and intergroup rivalries and individual’s role as fans (Lock and Heere, 2017), they also have faced criticism. Chief among these criticisms is that the social identity is primarily a cognitive construct. Ashforth and Mael (1989, p. 21) specifically noted that “identification is viewed as a perceptual cognitive construct that is not necessarily associated with any specific behaviors or affective states.” In the same vein, Foote (1951) indicated that affective state should be considered an antecedent or consequence of social identity. SIT’s emphasis on cognition can undermine its ability to explain the emotional dimensions of sport fandom fully. For instance, the intense joy of victory, the heartbreak of defeat, and the catharsis of collective celebration or mourning are central to the fan experience (Hirt et al., 1992). These emotions often transcend mere cognitive categorization, suggesting a need to expand beyond SIT’s theoretical boundaries.

Empirical studies further underscore the importance of emotions in sport fandom. Wann et al. (2001) found that emotional involvement was a stronger predictor of fan satisfaction and loyalty than cognitive identification. Similarly, Yim and Byon (2018) demonstrated that the emotional intensity of game experiences influenced fans’ willingness to invest time and resources in their team. These findings suggest that incorporating emotional dimensions into the study of sport fandom can provide a more holistic understanding of fan behavior. By acknowledging the interplay between cognitive and emotional processes, researchers can better explain phenomena such as enduring loyalty, intense rivalries, and the cathartic nature of fan rituals.

Identity fusion theory bridges the gaps in SIT and RIT by addressing both the cognitive and emotional dimensions of group membership. It explains how individuals experience a sense of oneness with a group, creating a fusion of personal and group identities (Swann et al., 2009). Unlike SIT, IFT preserves the personal identity within the group dynamic, allowing for both individuality and group identification to coexist. Unlike RIT, IFT emphasizes the dynamic, emotional ties that motivate behaviors, particularly in high-stakes or extreme scenarios.

One of the most significant advantages of IFT is its ability to explain extreme loyalty and self-sacrificial behaviors often found among sports fans. While SIT can account for ingroup favoritism, it does not fully capture why fans might go beyond normative behaviors to engage in acts of personal sacrifice. For instance, fans who feel fused with their sports team are more likely to prioritize the team’s success over their personal welfare, even in situations involving significant personal cost, such as traveling across continents for matches or enduring financial strain to attend games (Gómez et al., 2011). IFT also explains why fans engage in behaviors like defending their team in public or online forums, even in the face of criticism or opposition. Such actions are driven by a deep emotional investment and a sense of personal responsibility for the group’s welfare, which SIT and RIT may not be able to fully address.

#### Relational ties and collective ties

The study of sports fandom has been dominated by SIT (Tajfel and Turner, 1979), which explains how individuals derive a sense of self from their group affiliations, particularly their identification with a sports team. This framework has provided extensive insights into the cognitive processes underlying sports fan behaviors, such as in-group favoritism and out-group derogation (Lin and Bruning, 2020; Wann and Grieve, 2005). However, despite its prominence, SIT primarily emphasizes the psychological connection between fans and their favorite teams (Heere and James, 2007; Kwon et al., 2008; Kwon et al., 2022; Lock and Heere, 2017), often overlooking the social dynamics that influence fandom development and sustainability, particularly through interpersonal relationships within families, friend groups, and larger fan communities (Asada and Ko, 2019). Given that many individuals become sports fans due to socialization processes, including influence from significant others such as parents, friends, and other fans in communities (Asada and Ko, 2019; James, 1997), there is a need for a theoretical framework that better incorporates this kind of relational dimensions. Identity fusion theory (Swann et al., 2009) presents a promising solution to this limitation by addressing both relational ties and collective ties, offering a more holistic perspective on fan behavior.

Sports fandom is often cultivated through relational connections rather than solely through direct engagement with a team. Parental influence, in particular, has been identified as a key determinant of sports team affiliation, with studies indicating that children frequently adopt their parents’ team preferences (Spaaij and Anderson, 2012; Tinson et al., 2017). Similarly, peer influence plays a significant role in shaping fan identity, as individuals often become invested in a team due to social bonding experiences, such as attending games with friends or engaging in shared rituals (Spaaij and Anderson, 2012; Yim et al., 2021).

Émile Durkheim’s sociological framework provides a profound lens through which to understand sports fans’ relational connection, particularly emphasizing the role of collective rituals and shared emotional experiences. Central to Durkheim’s theory is the concept of collective effervescence, a phenomenon where individuals in a group experience a heightened sense of energy and unity during communal gatherings, leading to a reinforcement of social bonds and collective identity.

In the context of sports fandom, collective effervescence manifests vividly during events such as games, tailgates, and victory parades. These gatherings transcend mere entertainment; they become ritualistic ceremonies where fans engage in synchronized chants, wear team colors, and partake in shared traditions. Such activities foster a sense of belonging and communal identity, effectively transforming individual spectators into a cohesive unit. As Durkheim posited, these collective rituals are instrumental in reinforcing the collective conscience—the shared beliefs and values that bind a community together (Durkheim, 1995). Beyond the theoretical contribution of IFT, recent work on collective rituals and emotional synchrony further illuminates the relational and affective mechanisms that underlie extreme fan behavior. From a renewed Durkheimian vantage, collective rituals do more than express shared identity; they generate powerful waves of emotional synchrony and bonding (Rimé and Páez, 2023). These effervescent moments—such as Liverpool supporters joining voices in “You’ll Never Walk Alone” at Anfield or Hanshin Tigers fans performing their signature sixth-inning dance in perfect unison—can catalyze identity fusion through relational emotions like kama muta, the profound “moved by love” sensation arising when communal closeness intensifies suddenly (Zabala et al., 2024).

This ritual-based affect aligns with our emphasis on relational ties within fan communities. While SIT traditionally stresses depersonalized group membership and IFT foregrounds the merging of personal and social selves, these sporting rituals show how individuals come to feel viscerally and personally bound to fellow fans. The kama muta triggered by synchronized chants, collective goal celebrations, or victory parades reinforces the perception of the group not merely as a social category but as an emotionally meaningful community (Zabala et al., 2023).

Moreover, these insights resonate with elaborations of SIT—particularly the Elaborated Social Identity Model (ESIM)—which underscore how intergroup dynamics and legitimacy appraisals shape emotional engagement and relational transformations within crowds (Drury and Reicher, 2000, 2020). ESIM’s dynamic account helps explain why, for example, perceived unfair officiating can transform routine fandom into coordinated protest or pitch invasion. While IFT provides a precise framework for interpreting extreme, self-sacrificial behaviors, it attends less to these situational, legitimacy-driven processes. Recognizing the role of collective rituals, emotional synchrony, and ESIM’s processual perspective thus supports an integrative theoretical approach—one that bridges cognition, emotion, and context rather than casting SIT and IFT in binary opposition (Hopkins et al., 2016; Neville and Reicher, 2011). These relational dynamics highlight the importance of considering how sports fans construct and maintain their identities not only in relation to a team but also through meaningful connections with other fans.

As noted above, despite the extensive application of SIT in fan studies, it fails to fully capture the depth of these relational bonds. The theory primarily conceptualizes group membership as a cognitive categorization process in which individuals align themselves with in-groups and distinguish themselves from out-groups (Tajfel and Turner, 1979). While this perspective is useful in explaining phenomena such as team identification, team loyalty, and team licensed merchandize consumption (Kwon and Armstrong, 2002; Kwon et al., 2022), it does not adequately address the intensity of interpersonal relationships among fans. Although Woo et al. (2009) tried to incorporate diverse aspects of fans’ psychological attachment (i.e., team, coach, university, and players), they also overlooked the importance of other fans in their model. However, research has shown that many sports fans experience a profound sense of belonging not just to the team but also to fellow supporters, forming tight-knit communities characterized by shared rituals, traditions, and emotional connections (Guschwan, 2018). These findings suggest that fandom is not merely a matter of categorization but also of deep, personal commitment to a social network.

Identity fusion theory provides a theoretical framework that integrates both relational ties and collective identity, offering a more comprehensive understanding of sports fan behavior. This theory posits that individuals can experience an extreme form of identity alignment in which personal and group identities become functionally equivalent, leading to strong commitments to both the group and individual members (Swann et al., 2009). Unlike SIT, which views group membership as a depersonalized process, identity fusion theory emphasizes the emotional and relational dimensions of group attachment, recognizing that individuals can feel personally and emotionally bonded to other members of a group in addition to the group as a whole. This perspective has been used to explain intense forms of group loyalty, including the willingness to make personal sacrifices for the group (Swann et al., 2012). Applied to sports fandom, this suggests that fans who experience identity fusion not only support their teams with unwavering loyalty but also develop strong interpersonal relationships with other fans, reinforcing their commitment to the group.

Empirical research on identity fusion in sports contexts further supports this theoretical shift. Studies have found that ultra-fans, particularly those involved in organized supporter groups, often exhibit behaviors consistent with identity fusion, such as engaging in collective rituals, chants, and acts of group defense (Newson et al., 2016). These behaviors suggest that fandom is not merely a cognitive category but an embodied, emotional experience that connects fans both to their teams and to each other. Additionally, research on stadium environments has shown that fans who regularly attend live games report stronger relational bonds with fellow spectators, indicating that the physical and social context of fandom can reinforce both relational and collective identity (Gantz, 2013). A recent meta analysis on IFT also supports the above argument. Varmann et al. (2024) offered the first comprehensive meta-analytic examination of IFT and its relationship with extreme pro-group behaviors. Drawing on 90 studies, they noted that identity fusion demonstrates a strong and positive correlation (average *r* ≈ 0.50) with extreme pro-group orientations. Compared to social identification, identity fusion is generally a stronger predictor of extreme pro-group outcomes such as willingness to fight, die, or engage in violent collective actions (Varmann et al., 2024).

By incorporating identity fusion theory into the study of sports fandom, researchers can better explain the depth of commitment exhibited by fans, particularly in relation to the social structures that sustain fandom over time. This approach acknowledges that fans are not only attached to their teams but also to the people with whom they share their fan experiences, whether in families, friendships, or stadium communities. Given the increasing importance of social networks in shaping fan identities, future research should explore how identity fusion manifests in different fan cultures and how it interacts with traditional social identity processes. Ultimately, integrating identity fusion theory into the study of sports fandom allows for a more nuanced understanding of the social and emotional bonds that define fan communities, bridging the gap between individual and group-based explanations of fan behavior.

## Discussion

This paper explores the applicability of IFT in explaining sports fan behaviors and highlights its potential advantages over SIT and RIT. While SIT and RIT have contributed significantly to understanding group behavior and identity, they exhibit notable limitations in capturing the emotional, relational, and behavioral complexities of sports fandom. In particular, SIT effectively explains group-level identification and the pride fans derive from associating with successful teams (Tajfel and Turner, 1979; Chun and Sagas, 2022). However, it struggles to explain the deep emotional and personal loyalty fans maintain during periods of team failure. Similarly, RIT sheds light on the performative aspects of fandom, such as attending games or wearing licensed merchandize (Burke and Tully, 1977), but does not adequately address the emotional and relational depth of fans’ identities, where teams are often described as “family.” These limitations highlight the need for a more comprehensive theoretical framework.

Identity fusion theory, developed by Swann et al. (2009), addresses these gaps by emphasizing the visceral sense of “oneness” individuals feel with a group, merging personal and group identities. This perspective captures the profound emotional connections, relational ties, and self-sacrificial behaviors often observed in sports fandom. Unlike SIT, which centers on cognitive processes, IFT focuses on emotional bonds formed through shared experiences, such as victories, losses, and rituals (Drury et al., 2009; Swann et al., 2012). Furthermore, IFT’s integration of relational and collective ties explains not only the strong personal relationships among fans but also their shared identity with the team as a whole (Swann et al., 2010).

Identity fusion theory also provides a robust explanation for extreme fan behaviors, such as enduring financial hardship, traveling long distances, or demonstrating unwavering loyalty despite adversity. This level of commitment stems from the fused identity that links fans’ sense of self with the team’s successes and failures. Additionally, IFT accounts for intense rivalries and acts of devotion, such as tattooing team symbols or naming children after players which SIT and RIT fail to adequately explain.

Recognizing that identity-fused fans perceive their personal identity as intertwined with the team’s identity, marketing strategies should aim to reinforce this bond by emphasizing narratives that highlight shared values, history, and collective achievements. For instance, campaigns that celebrate the team’s legacy and its impact on the community can resonate deeply with fused fans, enhancing their emotional investment. Additionally, personalized marketing efforts, such as targeted communications that acknowledge fans’ long-term support or significant milestones, can further solidify this connection.

To foster positive forms of identity fusion, fan engagement programs should prioritize inclusivity, recognition, and shared experiences. Implementing loyalty programs that reward consistent support, providing exclusive content or access to team events, and highlighting fan stories can validate fans’ dedication and enhance their connection to the team. Furthermore, leveraging digital platforms to facilitate interactions among fans and between fans and the team can create a vibrant community that supports positive engagement. It is also crucial to establish clear codes of conduct and promote respectful behavior to mitigate negative outcomes such as hooliganism. Educational initiatives that emphasize sportsmanship and the team’s commitment to positive values can guide fans towards constructive expressions of their identity fusion.

While IFT offers distinct advantages in explaining the emotional and relational depth of sports fandom, it is not without its limitations. A primary weakness of IFT, when compared to SIT, lies in its narrower empirical base and less established theoretical constructs. SIT, with decades of research and application, has been extensively validated across diverse contexts, offering a comprehensive framework for understanding group behavior. Its emphasis on cognitive processes, such as self-categorization and social comparison, provides a versatile lens for examining group dynamics, including in-group favoritism and intergroup conflict (Tajfel and Turner, 1979). In contrast, IFT is a relatively newer theory, with limited application beyond specific domains such as military groups (i.e., Hart and Lancaster, 2019; Theys et al., 2020) and extremist organizations (i.e., Seyle, 2007). This narrower scope raises questions about its generalizability and applicability to broader group behaviors including sports fans.

Another significant limitation of IFT is its heavy reliance on the concept of “oneness” and the fusion of personal and group identities. While this concept effectively explains extreme loyalty and self-sacrificial behaviors, it may oversimplify the complex motivations underlying group attachment. For instance, not all fans who exhibit strong emotional ties to a team necessarily experience identity fusion. SIT, by contrast, offers a more nuanced understanding of group membership, incorporating multiple levels of identification (e.g., subgroup identity, and superordinate group identity) (Lock and Funk, 2016; Hornsey, 2008). This multi-level framework allows SIT to account for a wider range of group behaviors, from casual affiliations to deeply entrenched loyalties, which IFT does not address as comprehensively. Yet, SIT tends to treat social identity as relatively static once activated and emphasizes cognitive processes—such as categorization and comparison—without fully accounting for how emotional engagement among in-group members shapes collective action. Drury and Reicher’s (2000, 2020) Elaborated Social Identity Model addresses these limitations by framing crowd behavior as guided by context-sensitive social identities infused with both cognitive and emotional dynamics. According to ESIM, individuals in crowds enact shared identities that are continually reshaped through encounters with rival groups and authorities, rather than succumbing to deindividuation (Drury and Reicher, 2000). By foregrounding emotional solidarity—how shared feelings of injustice or camaraderie bind fans together—ESIM supplements SIT’s cognitive focus and reveals how affective engagement amplifies collective responses. In the context of sports fandom, ESIM proves particularly valuable as it explains how fans who perceive provocations from opposing supporters or unfair rulings by referees reconstitute their “us” against a common “them,” generating novel norms and actions that SIT alone cannot predict. Moreover, ESIM emphasizes that fans’ judgments of the legitimacy of external actions—whether by league officials, security personnel, or referees—critically determine their collective reactions (Stott and Reicher, 1998). Perceived illegitimacy intensifies both cognitive identification and emotional solidarity, catalyzing coordinated protests, demonstrations, or even confrontational behavior. ESIM also offers a processual account of how peaceful support may escalate into unrest: external threats or injustices activate emergent group norms and affective bonds, which in turn bolster cohesion and prompt collective actions that transcend SIT’s largely static framework.

Additionally, IFT’s focus on intense emotional bonds and extreme behaviors may inadvertently marginalize more moderate forms of fandom. While it is well-suited to explaining highly committed fans who demonstrate self-sacrificial behaviors, it is less effective in accounting for the majority of fans who engage in less intense but still meaningful forms of support. SIT’s broader applicability allows it to capture these varying degrees of attachment, providing a more inclusive framework for understanding sports fandom.

Another notable weakness of IFT is its limited emphasis on intergroup dynamics compared to SIT. SIT’s foundational principle of in-group versus out-group categorization provides a robust framework for understanding intergroup conflict, rivalry, and prejudice (Tajfel and Turner, 1986). In the context of sports fandom, this aspect of SIT is particularly relevant, as rivalries and competition between fan groups are central to the sports experience. While IFT addresses the intensity of intra-group bonds, it does not provide a detailed explanation of how these bonds influence intergroup interactions. For example, SIT explains how in-group favoritism and out-group derogation emerge as mechanisms for maintaining positive social identity, offering insights into the dynamics of rivalries and aggression between fan groups (Brewer, 1999). In contrast, IFT’s primary focus on intra-group fusion limits its utility in analyzing these intergroup phenomena.

Lastly, the methodological challenges associated with measuring identity fusion present another limitation. While validated scales for measuring SIT-related constructs, such as organizational identification (Mael and Ashforth, 1992) and collective self-esteem (Luhtanen and Crocker, 1992) are widely available and extensively used, the measurement of identity fusion remains less standardized (Swann et al., 2009). The reliance on self-reported measures, such as the pictorial scale of identity fusion, raises concerns about reliability and validity, particularly when applied to diverse cultural and contextual settings. These methodological limitations hinder the ability of researchers to compare findings across studies and contexts, thereby constraining the theory’s broader applicability.

Future research should further explore the applicability of IFT in diverse contexts and examine its potential to inform interventions aimed at fostering positive fan behaviors while mitigating negative outcomes such as aggression and rivalry-related conflicts. Furthermore, applying the scale of IFT specifically to sports fans represents a critical next step in this area of research. While the principles of IFT have been extensively studied in other group contexts, its application to sports fans remains relatively underexplored. By utilizing validated IFT measurement scales, researchers can investigate whether the same patterns of identity fusion observed in other domains hold true for sports fandom. Such studies could provide deeper insights into the emotional and behavioral dynamics of fans and help refine the theoretical framework to better address the unique characteristics of sports-related identities. Exploring the extent to which IFT’s principles apply to sports fans would not only validate the theory in this domain but also pave the way for practical applications, such as enhancing fan engagement strategies and fostering healthier fan communities.

Future research could also explore the behaviors of hooligans through the lens of SIT and identity fusion theory to determine which framework better explains such extreme behaviors. Hooliganism, characterized by acts of violence, intense rivalries, and disruptive behavior, provides a compelling context for testing the explanatory power of SIT and IFT. While SIT might explain hooliganism as a function of in-group versus out-group dynamics and the pursuit of self-esteem through group-based superiority IFT offers a perspective rooted in the visceral sense of oneness and shared destiny that could motivate individuals to engage in such extreme acts (Swann et al., 2009). For example, hooligans often exhibit a profound sense of loyalty and self-sacrificial behavior toward their group, which aligns with the principles of identity fusion. Comparative studies could examine the predictive power of SIT and IFT in explaining hooligan behaviors, offering valuable insights into the psychological mechanisms underpinning these actions. Such research could also inform interventions aimed at reducing hooliganism by addressing its underlying motivational and identity-based drivers.
